# Longitudinal Relationships between Nomophobia, Addictive Use of Social Media, and Insomnia in Adolescents

**DOI:** 10.3390/healthcare9091201

**Published:** 2021-09-11

**Authors:** Chung-Ying Lin, Marc N. Potenza, Martin Ulander, Anders Broström, Maurice M. Ohayon, Vijay Kumar Chattu, Amir H. Pakpour

**Affiliations:** 1 Institute of Allied Health Sciences, College of Medicine, National Cheng Kung University, Tainan 701, Taiwan; cylin36933@gmail.com; 2 Department of Public Health, National Cheng Kung University Hospital, Tainan 704, Taiwan; 3 Departments of Psychiatry and Neuroscience and the Child Study Center, School of Medicine, Yale University, New Haven, CT 06510, USA; marc.potenza@yale.edu; 4 Connecticut Council on Problem Gambling, Wethersfield, CT 06109, USA; 5 Connecticut Mental Health Center, New Haven, CT 06519, USA; 6 Department of Clinical Neurophysiology, Linköping University Hospital, 58183 Linköping, Sweden; martin.ulander@liu.se (M.U.); anders.brostrom@ju.se (A.B.); 7 Department of Nursing, School of Health and Welfare, Jönköping University, 55333 Jönköping, Sweden; 8 Division of Public Mental Health and Population Sciences, Stanford Sleep Epidemiology Research Center, Stanford University, Palo Alto, CA 94303, USA; mohayon@stanford.edu; 9 Division of Occupational Medicine, Department of Medicine, Temerty Faculty of Medicine, University of Toronto, Toronto, ON M5C 2CS, Canada; 10 Department of Public Health, Saveetha Medical College, Saveetha Institute of Medical and Technical Sciences, Saveetha University, Chennai 600077, India; 11 Social Determinants of Health Research Center, Research Institute for Prevention of Non-Communicable Diseases, Qazvin University of Medical Sciences, Qazvin 34199-15315, Iran

**Keywords:** adolescence, addiction, internet, insufficient sleep, phobia, social networking, sleep problems

## Abstract

(1) Background: Temporal relationships between nomophobia (anxiety related to ‘no mobile phone phobia’), addictive use of social media, and insomnia are understudied. The present study aimed to use a longitudinal design to investigate temporal relationships between nomophobia, addictive use of social media, and insomnia among Iranian adolescents; (2) Methods: A total of 1098 adolescents (600 males; 54.6%; age range = 13 to 19) were recruited from 40 randomly selected classes in Qazvin, Iran. They completed baseline assessments. The same cohort was invited to complete three follow-up assessments one month apart. Among the 1098 adolescents, 812 (400 males; 49.3%; age range = 13 to 18) completed the baseline and three follow-up assessments. In each assessment, the participants completed three questionnaires, including the Nomophobia Questionnaire (NMP-Q), Bergen Social Media Addiction Scale (BSMAS), and Insomnia Severity Index (ISI); (3) Results: Multilevel linear mixed-effects regression analyses showed that participants demonstrated increased insomnia longitudinally over 3 months (B = 0.12 and 0.19; *p* = 0.003 and <0.001). Insomnia was associated with nomophobia (B = 0.20; *p* < 0.001) and addictive use of social media (B = 0.49; *p* < 0.001). Nomophobia and addictive use of social media interacted with time in associations with insomnia as demonstrated by significant interaction terms (B = 0.05; *p* < 0.001 for nomophobia; B = 0.13; *p* < 0.001 for addictive use of social media); (4) Conclusions: Both nomophobia and addictive use of social media are potential risk factors for adolescent insomnia. The temporal relationship between the three factors suggests that parents, policymakers, and healthcare providers may target reducing nomophobia and addictive use of social media to improve adolescents’ sleep.

## 1. Introduction

To promote healthy development, good sleep (including sleep quality, sleep length, and sleep efficiency) is important for youth because it is vital for better cognitive and emotional processing [1,2]. Better cognitive and emotional processing promotes appropriate social interaction and learning throughout developmental epochs. Sleep is also associated with better physical health, mental well-being, and overall quality of life [3,4,5]. Therefore, optimal sleep quality among children and adolescents has been advocated [6]. Given changes in digital technologies and their use, use of smartphones, tablets, and other devices may interfere with sleep, as evidenced by associations between internet addiction and poor sleep in a recent meta-analysis [7] and the negative associations between screen time use and sleep [8,9,10].

As adolescents may be particularly prone to engaging in risky behaviors or pushing boundaries, including with respect to staying up beyond parentally imposed bedtimes, interventions promoting good sleep hygiene behaviors for adolescents have been promoted [6,11], especially to help adolescents resist attractions of digital technologies. Among internet-related concerns, problematic social media use (also termed addictive use of social media [12] warrants attention associated with poor sleep behaviors [13,14,15]. However, most studies linking poor sleep and addictive use of social media are cross-sectional, thereby providing little insight into directionality. Moreover, given that sleep is a broad concept, we intend to focus on one type of sleep disorders (i.e., insomnia) in the present study. Specifically, insomnia is defined as the presence of sleep difficulties, including difficulties in falling or staying asleep [16]. Additionally, empirical evidence from existing cross-sectional studies supports the associations between insomnia and problematic social media use [17]. However, there is a lack of longitudinal studies to assess the associations between addictive use of social media and insomnia. Therefore, longitudinal designs are needed to examine whether addictive use of social media may lead to insomnia, insomnia to addictive use of social media, both, or neither. 

Addictive use of social media has been considered as a type of specific internet addiction [18,19]. Moreover, some scholars have found that addictive use of social media is entangled with smartphone addiction and some progress in the taxonomy in this field has been made [20]. Specifically, smartphone addiction is viewed as a generalized Internet addiction that may involve different types of specific Internet addictions [21]. Although no specific diagnostic criteria have been adopted for the addictive use of social media, addictive use of social media may constitute a behavioral addiction. The American Society of Addiction Medicine [22] recently defined addiction as “a treatable, chronic medical disease involving complex interactions among brain circuits, genetics, the environment, and an individual’s life experiences. People with addiction use substances or engage in behaviors that become compulsive and often continue despite harmful consequences”. A study by Musetti et al. has concluded that there is a positive association between a history of childhood emotional abuse (CEA) and problematic social networking site (SNS) use in a sample of Italian adolescents aged 13–19 years [23]. Another study by Musetti et al. among Italian adolescents found that increased time spent on the mobile phone, low reflective functioning scores, and high childhood trauma scores predicted Problematic Mobile Phone Use (PMPU) scores in the sample. The study further found that among males, PMPU was positively related to time spent on mobile phone and childhood traumatic experiences and negatively related to reflective functioning. In contrast, among females, PMPU was negatively associated with time spent on mobile phones for video gaming and with reflective functioning [24]. 

Internet gaming disorder has provisional diagnostic criteria in the latest edition of the Diagnostic and Statistical Manual of Mental Disorders (DSM-5) [25], and gaming disorder was included as a disorder due to addictive behaviors in the 11th revision of the International Classification of Diseases (ICD-11) [26]. According to core features of behavioral addictions, it is reasonable to anticipate that poor sleep may accompany poor control over repetitive, persistent, and dysfunctional use of social media. Thus, the addictive use of social media may lead to sleep problems [13,14,15]. Alternatively, individuals with insomnia or other sleep disturbances may awake at night and use the internet for social media usage. As such, insomnia may lead to the addictive use of social media.

Another important factor related to poor sleep is nomophobia, a psychological concern or anxiety highly related to not having access to smartphone use [27], and this may be due to the smartphone addiction mentioned above. The term nomophobia is derived from ‘no mobile phone phobia’ [28] and reflects technological changes in society [29,30]. Nomophobia was first defined with both mobile phone and computer use by King et al. [26]; later, King and colleagues [29] focused on mobile phone and Internet use, as depicted in the following passages. “Nomophobia is the modern fear of being unable to communicate through a Mobile Phone (MP) or the Internet. Nomophobia is a term that refers to a collection of behaviors or symptoms related to MP use. Nomophobia is a situational phobia related to agoraphobia and includes the fear of becoming ill and not receiving immediate assistance” [29]. In short, when individuals have nomophobia, they may suffer from greater levels of fear or anxiety because of being unable to engage in mobile phone usage. Thus, nomophobia may be viewed akin to a DSM-5-defined specific phobia [25] and has been related to addictive use of social media [28,30]. With phobia and addictive use of social media features, nomophobia may contribute to poor sleep (or the present study’s focus, insomnia). However, to the best of our knowledge, only one recent study using cross-sectional design reports the association between nomophobia and insomnia [31]. No additional studies, especially those with a longitudinal design, have examined relationships between nomophobia and sleep. Although nomophobia and addictive use of social media seem to be distinct constructs (i.e., nomophobia describes the fear of a perceived inability to access one’s smartphone; addictive use of social media describes a dependence on social media use), they may share associations with insomnia. Specifically, an underlying reason for nomophobia may be a dependence on social media use; thus, a person may crave using the social media functions via a smartphone (i.e., having a feature of addictive use of social media). Therefore, a person with nomophobia may have features of addictive use of social media. Indeed, the relationship between nomophobia and addictive use of social media has been reported [28,30]. As a result, nomophobia and addictive use of social media may share similar associations with insomnia. However, empirical evidence is needed to support this possibility. 

As evidence regarding the relationships between nomophobia, addictive use of social media, and insomnia is insufficient, the present study aimed to use a longitudinal design to investigate temporal relationships between nomophobia, addictive use of social media, and insomnia among Iranian adolescents. Based on existing data, two hypotheses were made. First, changes in levels of nomophobia would be associated with changes in insomnia severity, with increased nomophobia leading to greater insomnia. Second, changes in levels of addictive use of social media would be associated with changes in insomnia severity, with increased addictive use of social media leading to greater insomnia. In this context, the study aims to investigate temporal relationships between nomophobia, addictive use of social media, and insomnia among Iranian adolescents.

## 2. Materials and Methods

### 2.1. Participants and Procedure

Participants were adolescents aged between 13–19 years attending high schools in Qazvin (a city near Tehran), Iran. Adolescents were eligible to participate if they were 13–19 years old, spoke Persian, and owned a smartphone. Participants were recruited from a list of all high schools provided by the Qazvin Department of Education. Of 62 high schools, 40 were selected randomly. One class was then randomly selected from each school, and all adolescents of each class were invited to participate in the study. In a preliminary session, the participants were informed about the study. Eligible participants were then asked to read the consent form and provide written informed consent. In addition, consent forms from parents/caregivers/guardians were also obtained. All adolescents were asked to complete study measures on a monthly basis; at baseline (Time 1), 1 month later (Time 2), 2 months from baseline (Time 3), and 3 months from baseline (Time 4). No significant differences in gender distribution were found between participants who completed all assessments and those who did not (χ^2^ = 0.42; *p* = 0.52). 

### 2.2. Instruments

#### 2.2.1. Insomnia Severity Index

The Insomnia Severity Index (ISI) is a self-reported scale that uses seven 5-point Likert-scale items (scoring from 0 to 4) to assess insomnia severity [32,33]. The seven ISI item scores were summed, and a higher summated score indicates a more severe level of insomnia. Prior research has examined the psychometric properties of the Persian ISI and shown that it has satisfactory construct validity, concurrent validity, test-retest reliability, and internal consistency [34]. The internal consistency of the ISI in the present study was very good (α = 0.88). An ISI score of 9 or above indicates insomnia [34]. The Persian ISI used in the present study has been translated using standard methods [34]. An example item of the ISI is, “How satisfied/dissatisfied are you with your CURRENT sleep pattern?” 

#### 2.2.2. Nomophobia Questionnaire

The Nomophobia Questionnaire (NMP-Q) is a self-reported scale that uses 20 7-point Likert-scale items (scoring from 1 to 7) to assess nomophobia severity. The 20 NMP-Q item scores were summed, and a higher summated score indicates a more severe level of no-mophobia [35]. Prior research has examined the psychometric properties of the Persian NMP-Q and shown that it has satisfactory construct validity, concurrent validity, test-retest reliability, and internal consistency [27]. The internal consistency of the NMP-Q in the present study was very good (α = 0.91). An NMP-Q score of 21 or above indicates insomnia (https://www.psytoolkit.org/survey-library/nmp-q.html; accessed on 10 July 2021). The Persian NMP-Q used in the present study has been translated using standard methods [27]. An example item of the NMP-Q is, “I would feel uncomfortable without constant access to information through my smartphone”.

#### 2.2.3. Bergen Social Media Addiction Scale (BSMAS)

The Bergen Social Media Addiction Scale (BSMAS) is a self-reported scale that uses six 5-point Likert-scale items (scoring from 1 to 5) to assess the severity of addictive use of social media. The six BSMAS item scores were summed, and a higher summated score indicates a more severe level of addictive use of social media [12,36]. Prior research has examined the psychometric properties of the Persian BSMAS and shown that Persian BSMAS has satisfactory construct validity, concurrent validity, test-retest reliability, and internal consistency [37]. The internal consistency of the BSMAS in the present study was very good (α = 0.86). A BSMAS score of 19 or above indicates addictive use of social media [38]. The Persian BSMAS used in the present study has been translated using standard methods [37]. An example item of the BSMAS is, “How often during the last year have you felt an urge to use social media more and more?”

### 2.3. Statistical Analyses

Participants’ characteristics and NMP-Q, BSMAS, and ISI scores were first analyzed using descriptive statistics, including means with standard deviations and frequencies with percentages. Pearson correlations were conducted to understand associations between nomophobia (NMP-Q assessed), addictive use of social media (BSMAS assessed), and insomnia (ISI assessed) across the four-time points (i.e., baseline and three follow-up assessments). 

Linear mixed-effects regression analyses with three-level random intercepts [39] were used to assess longitudinal associations between (i) nomophobia and insomnia; and (ii) addictive use of social media and insomnia. Three models were proposed. The first model used nomophobia, nomophobia’s interaction with time, and covariates (age, gender, and father’s education) as independent variables. The second model used addictive use of social media, addictive use of social media’s interaction with time, and the same covariates as independent variables. The third model used nomophobia, nomophobia’s interaction with time, addictive use of social media, addictive use of social media’s interaction with time, and the same covariates as independent variables. All models adopted a three-level multilevel model, with measurements of insomnia at each time point (level 1) being nested within adolescents (level 2) who were in turn nested within schools (level 3), with random effects for students and schools to take into account dependency between repeated assessments and intracluster correlation between students in each school. Time-level, adolescent-level, and school-level variables were tested for significance, followed by significant interactions between significant variables, and finally, random slopes. Markov chain Monte Carlo methods with burn-in length 500 and monitoring chain length 5000 were used for model parameter estimations [40]. The Wald statistic was used to test statistically significant effects at *p* < 0.05 [41]. 

### 2.4. Ethics

Both parents and adolescents provided written informed consent before study participation. The study protocol was approved by the Ethics Committee of Qazvin University of Medical Sciences (n. IR.QUMS.REC.1398.416). 

## 3. Results

Over 70% of participants (n = 812 from 1098) completed baseline and all three follow-up assessments. Table 1 lists participant characteristics for the entire sample (600 [54.6%] males), those who completed all follow-ups, and those lost to follow-up(s). In brief, the participants, on average, spent 4.63 (SD = 2.88) hours on smartphones and 3.71 (SD = 2.12) hours on social media daily. The average sleep duration was 6.12 h and the average duration of smartphone use during the night was found to be 1.79 h. Table 2 additionally demonstrates participants’ levels of nomophobia, addictive use of social media, and insomnia across four-time points. The information of how many participants appeared at risk for nomophobia, addictive use of social media, and insomnia across four-time points are also illustrated in Table 2.

Correlations between nomophobia, addictive use of social media, and insomnia across four-time points were all significant (Table 3). Moreover, nomophobia was moderately correlated with the addictive use of social media (r = 0.416 to 0.614) and weakly to moderately correlated with insomnia (r = 0.218 to 0.366). Addictive use of social media was also weakly to moderately correlated with insomnia (r = 0.239 to 0.437).

The intraclass correlation coefficient was 0.102 for the multilevel linear mixed-effects regression model. These analyses showed that participants demonstrated more severe insomnia over time (B = 0.12 and 0.19; *p* = 0.003 and <0.001). Moreover, insomnia levels were associated with nomophobia (B = 0.20; *p* < 0.001) and addictive use of social media (B = 0.42 to 0.49; *p* < 0.001). Nomophobia and addictive use of social media interacted with time in associations with insomnia as demonstrated by significant interaction terms (B = 0.04 to 0.05; *p* < 0.001 for nomophobia; B = 0.10 to 0.13; *p* < 0.001 for addictive use of social media) (Table 4).

## 4. Discussion

The present study recruited a large sample of Iranian adolescents to investigate temporal relationships between nomophobia, addictive use of social media, and insomnia. The present findings support the two hypotheses that (i) changes in levels of nomophobia are associated with changes in insomnia severity and greater nomophobia is associated with more severe insomnia; (ii) changes in levels of addictive use of social media are associated with changes in insomnia severity, and greater addictive use of social media is associated with more severe insomnia. Insomnia severity increased from baseline to the 3-month follow-up period. The effects of nomophobia and addictive use of social media on insomnia appeared greater from baseline to the 3-month follow-up. Implications are discussed below. 

The relationship between nomophobia and insomnia found in the present study may relate to anxious or phobic features of nomophobia [25], as nomophobia is characterized by worry about missing social media interaction [29,30]. Anxiety is a type of psychological distress often associated with other types of distress, such as stress and depression [37]. Thus, nomophobia could be associated with psychological distresses that may lead to sleep problems. Prior research in adolescents indicates that psychological distress is associated with insufficient sleep or sleep disturbances [42,43]; therefore, adolescents may have insomnia resulting from nomophobia-induced psychological distress. Another possible explanation is that nomophobia promotes worry of missing social media interactions. Therefore, individuals may be in a stimulated and excited state, promoting insomnia. 

The relationship between addictive use of social media and insomnia may be explained by mechanisms proposed by prior research [15,44,45,46,47]. First, the use of social media is often an ongoing process; that is, there are no clearly defined beginnings and endings for social media interactions [15]. Indeed, some individuals have difficulties resisting responding in ways that may interfere with sleep. Social media interactions may also have a “waiting” feature (i.e., sending out messages and waiting for responses). Therefore, sleep patterns may be disrupted when an individual with potential addictive use of social media is preoccupied and waiting for replies from others [45]. Second, as an individual may wait for social interactions during normal sleep hours, bedtime may be delayed, leading to sleep rhythm desynchronization [44]. Such desynchronization may jeopardize sleep quality and induce insomnia. Third, light-emitting screens in the smartphone may suppress sleep-promoting hormones (e.g., melatonin that is typically elevated before bedtime) and disrupt sleep patterns [46]. Lastly, social media use may result in psychological stimulation; that is, an individual’s mood may become elevated and excited by social media interactions, and these emotional states may interfere with sleep [46,47]. 

The present study showed that the impacts of nomophobia and addictive use of social media on insomnia increased over time. The findings suggest the importance of early detection and interventions targeting nomophobia and addictive use of social media in adolescents. Healthcare providers, educators, parents, and other stakeholders should be curious about understanding nomophobia and addictive use of social media in youth, especially given that features of nomophobia and addictive use of social media may result in sleep problems and subsequent concerns for adolescents over time. Moreover, healthcare providers may consider implementing effective programs promoting sleep hygiene information on nomophobia and addictive use of social media. As developing good sleep hygiene behaviors has been linked to better sleep quality [6], the current findings suggest further consideration of specific aspects of internet use on sleep health. 

### Limitations

Limitations of the present study should be noted. First, the sample is restricted to Iranian adolescents. Additionally, the recruitment procedure is convenience sampling in one city of Iran. Therefore, the generalizability of the present study’s findings is limited. Second, all instruments were self-administered by the adolescents. Therefore, common method variance, together with recall bias and social desirability, cannot be avoided. The common method variance may lead to overestimation of associations among the studied variables, recall bias may lead to inaccurate responses, and social desirability may, for example, lead to underreporting of nomophobia, addictive use of social media, and insomnia. However, the present study showed strong psychometric properties of the three questionnaires on nomophobia, addictive use of social media, and insomnia; therefore, the impacts of this limitation on the present study’s findings may be limited.

Nevertheless, future studies should consider using objective measures of insomnia and digital technology to obtain more objective information. Third, other potential confounding factors such as the assessment of gaming disorder and other potentially problematic online activities (gambling, pornography viewing) were not collected. Other specific internet addictions and general internet addictions have been associated with sleep disturbances [7,13,15]. Furthermore, people with addictive use of social media often exhibit other behavioral addictions [48]. Therefore, the relationships between nomophobia, addictive use of social media, and insomnia found in the present study may be influenced by other types of behavioral addictions. Similarly, other formal psychiatric disorders (mood, anxiety, and substance use disorders) were not assessed, and future studies are thus needed to further clarify their potential contributions and relationships. 

## 5. Conclusions

In conclusion, the present study shows that both nomophobia and addictive use of social media are potential risk factors for adolescent insomnia. The present study seems to be the first to longitudinally investigate relationships between nomophobia, addictive use of social media, and insomnia among adolescents. Healthcare providers and others should consider the importance of reducing nomophobia and addictive use of social media in adolescents to improve their sleep. 

## Figures and Tables

**Table 1:** Participant characteristics.

	Entire Sample (N = 1065–1098)	Sample Completing All Follow-Ups (n = 762–812)	Sample with One Lost Follow-Up (n = 79–108)	Sample with Two Lost Follow-Ups (n = 68–88)	Sample with Three Lost Follow-Ups (n = 91–110)
Age	15.55 (3.21)/N = 1098	15.44 (3.33)/n = 812	15.38 (3.78)/n = 98	15.51 (4.12)/n = 88	15.46 (3.29)/n = 100
Gender (Male)	600 (54.6%)/N = 1098	443 (55.2%)/n = 802	52 (48.1%)/n = 108	49 (55.7%)/n = 88	56 (56.0%)/n = 100
Time on smartphone (hours/day)	4.63 (2.88)/N = 1083	4.57 (2.69)/n = 801	4.66 (2.81)/n = 108	4.53 (2.66)/n = 68	4.51 (3.19)/n = 106
Time on social media (hours/day)	3.71 (2.12)/N = 1078	3.68 (2.20)/n = 792	3.75 (2.39)/n = 106	3.60 (2.87)/n = 82	3.62 (2.70)/n = 98
Current smoker (No)	906 (84.0%)/N = 1079	649 (82.9%)/n = 783	94 (87.0%)/n = 108	76 (86.4%)/n = 88	87 (87.0%)/n = 100
Father’s education years	6.29 (2.91)/N = 1065	6.31 (2.48)/n = 795	6.33 (3.05)/n = 79	6.25 (2.89)/n = 83	6.30 (2.91)/n = 108
Body mass index (kg/m^2^)	21.34 (3.11)/N = 1065	21.28 (2.70)/n = 762	21.12 (2.77)/n = 108	21.01 (2.69)/n = 85	21.37 (2.48)/n = 110

**Table 2:** Nomophobia, social media addiction, and insomnia across time.

	Mean (SD)			
	Time 1	Time 2	Time 3	Time 4
Nomophobia	76.81 (11.71)	75.50 (10.15)	75.73 (11.21)	76.17 (12.36)
Addictive use of social media	14.55 (5.08)	13.96 (5.15)	14.53 (5.24)	14.60 (5.11)
Insomnia	8.53 (5.44)	8.12 (4.29)	8.47 (4.84)	8.66 (4.96)
	N (%) ^a^			
Nomophobia	533 (48.5%)	576 (53.9%)	571 (54.2%)	553 (52.1%)
Addictive use of social media	234 (21.3%)	226 (21.2%)	221 (21.0%)	240 (22.6%)
Insomnia	451 (41.1%)	419 (39.2%)	427 (40.5%)	462 (43.5%)

Note: Nomophobia was assessed by the Nomophobia Questionnaire (NMP-Q); social media addiction was assessed by the Bergen Social Media Addiction Scale; Insomnia was assessed by the Insomnia Severity Index. Time 1 = baseline; Time 2 = first follow-up; Time 3 = second follow-up; Time 4 = third follow-up. ^a^ number of participants at risk of nomophobia (cutoff ≥ 21), addictive use of social media (cutoff ≥ 19), and insomnia (cutoff ≥ 9).

**Table 3:** Correlation matrix among nomophobia, addictive use of social media, and insomnia across time.

	r											
	NMP-Q_T1	NMQ-Q_T2	NMP-Q_T3	NMP-Q_T4	BSMAS_T1	BSMAS_T2	BSMAS_T3	BSMAS_T4	ISI_T1	ISI_T2	ISI_T3	ISI_T4
NMP-Q_T1	--											
NMP-Q_T2	0.857	--										
NMP-Q_T3	0.782	0.638	--									
NMP-Q_T4	0.627	0.750	0.744	--								
BSMAS_T1	0.571	0.526	0.467	0.535	--							
BSMAS_T2	0.526	0.599	0.416	0.508	0.872	--						
BSMAS_T3	0.489	0.442	0.605	0.485	0.842	0.723	--					
BSMAS_T4	0.561	0.517	0.453	0.614	0.728	0.831	0.776	--				
ISI_T1	0.301	0.271	0.241	0.288	0.351	0.309	0.277	0.320	--			
ISI_T2	0.289	0.366	0.226	0.270	0.318	0.310	0.239	0.284	0.787	--		
ISI_T3	0.253	0.218	0.355	0.237	0.300	0.256	0.345	0.269	0.790	0.777	--	
ISI_T4	0.304	0.285	0.266	0.362	0.371	0.332	0.284	0.437	0.779	0.736	0.760	--

Note: NMP-Q = nomophobia, assessed by the Nomophobia Questionnaire; BSMAS = social media addiction, assessed by the Bergen Social Media Addiction Scale; ISI = insomnia, assessed by the Insomnia Severity Index; T1 = Time 1 (baseline); T2 = Time 2 (first follow-up); T3 = Time 3 (second follow-up); T4 = Time 4 (third follow-up). All coefficients’ *p*-values < 0.01.

**Table 4:** Results of the multilevel mixed model analyses with Insomnia Severity Index (ISI) total score as a dependent variable.

	Effects of NMP-Q on ISI			Effects of BSMAS on ISI			Effects of NMP-Q and BSMAS Simultaneously on ISI		
	B	SE	*p* (95%CI)	B	SE	*p* (95%CI)	B	SE	*p* (95%CI)
Time	0.12 **	0.04	0.003 (0.04, 0.20)	0.19 ***	0.05	<0.001 (0.09, 0.29)	0.12 **	0.04	0.003 (0.04, 0.20)
NMP-Q	0.20 ***	0.007	<0.001 (0.19, 0.21)	--	--	--	0.20 ***	0.007	<0.001 (0.19, 0.21)
BSMAS	--	--	--	0.49 ***	0.01	<0.001 (0.47, 0.51)	0.42 ***	0.05	<0.001 (0.32, 0.52)
Age	0.36 **	0.12	0.003 (0.12, 0.60)	0.31 **	0.10	0.002 (0.11, 0.51)	0.32 **	0.12	0.008 (0.08, 0.056)
Gender (Ref: female)	−0.90 **	0.30	0.003 (−1.49, −0.31)	−0.63 **	0.24	0.009 (−1.10, −0.16)	−0.84 *	0.33	0.011 (−1.49, −0.19)
Father’s education	0.04	0.04	0.32 (−0.04, 0.12)	0.03	0.05	0.55 (−0.07, 0.13)	0.01	0.02	0.62 (−0.03, 0.05)
NMP-Q × Time	0.05 ***	0.009	<0.001 (0.03, 0.07)	--	--	--	0.04 ***	0.009	<0.001 (0.02, 0.06)
BSMAS × Time	--	--	--	0.13 ***	0.03	<0.001 (0.07, 0.19)	0.10 ***	0.003	<0.001 (0.09, 0.11)
Intercept	1.92 ***	0.12	<0.001 (1.68, 2.16)	3.35 ***	0.62	<0.001 (2.13, 4.57)	1.81 ***	0.15	<0.001 (1.52, 2.10)
σ^st2 (adolescents)	19.34 ***	0.85	<0.001 (17.67, 21.01)	19.44 ***	0.84	<0.001 (17.79, 21.09)	20.05 ***	0.51	<0.001 (19.05, 21.05)
σ^sc2 (School)	2.19 ***	0.05	<0.001 (2.09, 2.29)	1.44 ***	0.04	<0.001 (1.36, 1.52)	2.34 ***	0.06	<0.001 (2.22, 2.46)

Note: * *p* < 0.05; ** *p* < 0.01; *** *p* < 0.001. NMP-Q = Nomophobia Questionnaire; BSMAS = Bergan Social Media Addiction Scale; ISI = Insomnia Severity Index.

## Data Availability

The datasets used for this research on adolescents cannot be shared with the public as per the privacy policy and regulations of Qazvin University of Medical Sciences.
